# The Impact of Autism Spectrum Disorder Severity on the Incidence and Extent of Traumatic Dental Injuries in Children: A Retrospective Observational Cohort Study

**DOI:** 10.1111/edt.70080

**Published:** 2026-05-07

**Authors:** Parisa Ghanouni, Pierre‐Luc Michaud, Annika Nobbe, Isabel Mello, Shannon Fitzpatrick, Erin Quigley, Jarrett Miller, Tracy Doyle

**Affiliations:** ^1^ IWK Health Centre Halifax Canada; ^2^ Department of Occupational Therapy, Faculty of Health Dalhousie University Halifax Canada; ^3^ Department of Pediatrics, Faculty of Medicine Dalhousie University Halifax Canada; ^4^ Department of Dental Clinical Sciences, Faculty of Dentistry Dalhousie University Halifax Canada

**Keywords:** autism, children, retrospective chart review, severity of disorder, traumatic dental injury

## Abstract

**Introduction:**

Traumatic dental injuries are a common oral health problem among children and have significant health consequences. Children with autism spectrum disorder may face an increased risk of traumatic dental injuries, potentially due to developmental delays, sensory difficulties, and behavioral challenges. However, the relationship between the severity of autism and the extent and occurrence of dental trauma remains unclear. To our knowledge, this is the first study to aim at evaluating the association between the severity of autism and the incidence and severity of dental trauma.

**Methods:**

This retrospective chart review analyzed data from children aged 3–15 years with autism spectrum disorder, who received their health and dental care at a major pediatric health center between 2016 and 2024. A total of 441 patients with autism were included in the study. Statistical analyses were used to examine associations between sex, autism severity, and dental trauma incidence and type/severity.

**Results:**

Among the 441 participants, 9.1% experienced at least one dental trauma. No statistically significant associations were found between autism severity and dental trauma incidence (*p* = 0.136) or severity (*p* = 0.517). Furthermore, sex was not associated with the occurrence of dental trauma (*p* = 0.591).

**Conclusion:**

The severity of autism spectrum disorder and sex are not associated with the occurrence or severity of dental trauma among children. These findings suggest that factors beyond the severity of autism may play a more significant role in the risk and impact of dental trauma in this population. A clearer understanding of additional contributing variables is essential to inform more effective prevention and management strategies for high‐risk individuals within this population.

## 1 Introduction

Traumatic dental injuries (TDI) represent a major public health concern due to their high prevalence, particularly among children and adolescents, their long‐term functional and psychosocial consequences, and the substantial economic burden associates with their treatment [1]. TDI refers to traumatic injuries affecting the teeth and surrounding hard and soft tissues in the oral cavity [1, 2]. The severity of TDI is classified based on the tissue type involvement and their extent, ranging from injuries affecting soft tissues only, such as lacerations or bruising of the gingiva and lips, to hard tissue damage, including tooth and bone fractures and injuries to periodontal tissues [3, 4]. While hard tissue injuries such as root fractures or tooth avulsions are more severe, periodontal injuries are more frequently observed in accidents. In periodontal injuries, mild cases (e.g., concussions and subluxations) involve tenderness or increased tooth mobility without significant displacement; moderate injuries (e.g., lateral luxation and extrusion) require tooth repositioning or splinting; and severe injuries (e.g., avulsion and root fracture) result in more profound consequences [5]. Crown fractures can also vary in severity, depending on restorability and pulp exposure [4]. TDI is most likely to occur during early childhood, with rates gradually decreasing with age. The effects of TDI in childhood can be long‐lasting, as injuries during tooth formation may potentially cause the malformation of permanent teeth [6].

Previous systematic reviews indicate that individuals with autism spectrum disorder (ASD) may show a higher risk of TDI compared to neurotypical populations [7, 8]. ASD is a common neurodevelopmental disorder characterized by restricted and repetitive behaviors and difficulties in social interactions [9]. Research on TDI in children with ASD is limited, hindering understanding of local risk factors and the development of tailored prevention and care. Despite a higher prevalence of TDI in children with ASD and the substantial oral health and economic burden associated with these injuries [7, 8, 10, 11, 12, 13, 14, 15], no studies have specifically explored the underlying reasons for this increased risk. Some have suggested that ASD severity may be linked to behaviors that could predispose individuals to TDI, but this relationship has not been systematically investigated.

ASD severity is medically categorized into three levels, reflecting the child's varying support needs. Level 1 individuals generally live independently with minimal support and can communicate verbally, though social challenges exist. Level 2 requires substantial support for communication and daily activities, with more pronounced social and verbal difficulties. Level 3 individuals need very substantial support, often have limited speech and rely heavily on others for daily living and social interactions [16]. ASD severity, as reflected in the three support levels, is a key indicator of a child's needs in daily activities and dental care. Children with higher support needs (levels 2–3) often face challenges during dental visits, including adapting to unfamiliar environments. They may also struggle to maintain oral hygiene and are more likely to experience dental caries, gingival bleeding, and irregular dental check‐ups compared with their peers [10, 17].

A higher level of ASD severity may also be associated with an increased risk of more frequent or severe TDI due to greater motor or cognitive impairments, reduced awareness of environmental hazards, sensory processing challenges, and higher rates of self‐injurious or risk‐taking behaviors [7, 11]. Unlike neurotypically developing individuals, whose dental injuries often occur during group or leisure activities, individuals with ASD may experience TDI from daily movements [18]. Self‐injurious behaviors (e.g., head banging or hitting their face) are more common in severe ASD and may arise from agitation, anxiety, emotion dysregulation, or sensory processing difficulties, potentially increasing the risk of TDI [18, 19].

Although children with ASD may be at a higher risk of TDI compared to their typically developing peers, the influence of ASD severity and the underlying mechanisms remain unclear. Understanding the association between ASD severity and the incidence and severity of TDI, as well as identifying contributing risk factors, may help guide the development of targeted interventions and preventive strategies to address the unique dental needs of children with ASD and reduce the incidence and severity of dental trauma. As it is hypothesized that higher ASD severity is associated with an increased frequency and severity of TDI, the aim of this study was to evaluate the possible association between ASD severity and the incidence and severity of TDI.

## 2 Method

### 2.1 Study Design

This study employed a retrospective observational cohort design, consisting of a chart‐based review of data spanning 8.5 years at a major pediatric health center in Atlantic Canada. This center provides care for patients across the full spectrum of ASD severity, from mild to severe, and serves families from all Atlantic Canadian provinces, including Nova Scotia, New Brunswick, Prince Edward Island, and Newfoundland and Labrador. The center's broad service area and annual patient volume encompass a geographically and clinically diverse population, enhancing the robustness and generalizability of the data generated. This study was reported in accordance with the STROBE guidelines [20].

Data were extracted from medical and dental records and analyzed to evaluate associations and rates in this population. The project received approval from the institutional Research Ethics Board prior to debuting the study. Due to the retrospective design of the study, its large sample size, and its non‐interventional nature with minimal risk, a waiver of informed consent was approved by the Research Ethics Board.

### 2.2 Study Population

Medical and dental records were retrieved for all patients with ASD aged 3 to 15 years, who received medical and dental treatments at the health center between January 2016 and June 2024. This age range was selected because ASD is commonly diagnosed after the age of three, and dental injuries are more frequent among children under 15. Access to both medical and dental records enabled comprehensive and reliable data collection for the study population.

The inclusion criteria were patients diagnosed with ASD and aged 3–15, who received medical and dental care within the specified timeframe. Children diagnosed with ASD after January 2024 were excluded to ensure a minimum observation period of 6 months following diagnosis, providing sufficient time for a traumatic dental injury to occur. In addition, patients with limited medical chart information that prevented confirmation of their ASD diagnosis were also excluded.

Two research assistants with dental backgrounds were trained together to ensure consistency and independently extracted data from dental and medical charts. Prior to the start of independent data collection, they jointly reviewed several dental and medical charts to ensure standardization in the data gathering process. Medical and dental charts from all patients included in the study were reviewed independently by a research assistant. For each patient, both the medical and dental charts were reviewed by the same assistants. Medical records were examined to determine each child's date of ASD diagnosis and its severity level. Classification of severity level was based on documented descriptions of social, communicative, and functional abilities in accordance with the Diagnostic and Statistical Manual of Mental Disorders [16]. Dental records were reviewed to retrieve date of birth, sex, type/severity of TDI including fractures (enamel, enamel‐dentin, enamel‐dentin‐pulp, crown‐root, and root), concussion, subluxation, luxation (lateral, intrusive, extrusive), and avulsion based on the International Association of Dental Traumatology guidelines [21]. Given the challenges in comparing the severity of soft‐tissue and hard‐tissue injuries, the type of traumatic dental injury was used as a surrogate for TDI severity, employing nominal variables rather than ordinal ones.

### 2.3 Data Analysis

Statistical analyses were performed using a statistical software program (IBM SPSS Statistics, version 29; IBM Corp.), with statistical significance set at *p* < 0.05. To examine the relationship between sex and the incidence of TDI, a Chi‐Square test of independence was conducted. To assess the association between ASD severity and the development of TDI, a Chi‐Square test of independence was also used. In addition, to evaluate whether the likelihood of developing a TDI increased with ASD severity, a Mantel–Haenszel test for linear trend was conducted. This test was chosen to assess a monotonic relationship across the ordered ASD severity levels. Lastly, to examine the association between ASD severity and the severity of TDI, a Fisher–Freeman–Halton exact test of independence was performed. This test was selected because some cells of the contingency table had low expected frequencies, making the standard chi‐square test inappropriate.

## 3 Results

A total of 452 patients meeting the inclusion criteria were identified. Of these, 8 were excluded due to the absence of an ASD diagnosis date, 2 were excluded because insufficient chart information prevented confirmation of the ASD diagnosis, and 1 patient was excluded because the ASD diagnosis occurred after January 2024. As a result, 441 ASD participants were included in the study, comprising 335 males (76%) and 106 females (24%). Of these, 40 individuals (9.1%), including 29 males and 11 females, experienced at least one incident of TDI, while 401 (306 males and 95 females) did not. The types of TDI encountered are summarized in Table 1. A Chi‐Square test of independence was conducted to examine the relationship between sex and the occurrence of TDI. The association was not statistically significant, *χ* ^2^(1, *N* = 441) = 0.289, *p* = 0.591, indicating that sex was not associated with TDI occurrence.

**TABLE 1:** Types of traumatic dental injury (TDI) encountered.

Type of TDI	Frequency
Fracture limited to enamel	2 (5%)
Fracture extending to dentin	11 (27.5%)
Fracture with pulp exposure	1 (2.5%)
Fracture Crown to root	1 (2.5%)
Concussion	13 (32.5%)
Subluxation	3 (7.5%)
Subluxation with dentinal fracture	1 (2.5%)
Extrusive or lateral luxation	4 (10%)
Extrusive or lateral luxation with dentinal fracture	1 (2.5%)
Avulsion	3 (7.5%)

Analyses were also conducted to assess the relationship between ASD severity and the incidence and severity of TDI. Of the 441 ASD participants, 437 had complete data on ASD severity and were included in these analyses. Among them, about 15.9% of participants required support, 51.2% required substantial support, and 32% required very substantial support. TDI occurred in 2.9% of participants requiring support (2/70), 10.6% requiring substantial support (24/226), and 9.2% requiring very substantial support (13/141). Table 2 shows a detailed breakdown of ASD severity among participants who developed a TDI and those who did not. A Chi‐Square test of independence found no significant association between ASD severity level and the presence of TDI, *χ* ^2^(2, *N* = 437) = 3.984, *p* = 0.136. Similarly, the Mantel–Haenszel test for linear trend revealed no significant monotonic relationship between increasing ASD severity and the likelihood of developing a TDI, *χ* ^2^(1, *N* = 437) = 1.338, *p* = 0.247. These results suggest that ASD severity does not significantly influence whether a TDI occurs. Finally, a Fisher–Freeman–Halton exact test of independence was conducted to examine the association between ASD severity and TDI severity. The test was not statistically significant, *p* = 0.517, indicating no meaningful association between ASD severity and the type/severity of TDI sustained. Table 3 presents the distribution of TDI type/severity in relation to the severity of ASD.

**TABLE 2:** Severity of autism spectrum disorder (ASD) among participants with and without traumatic dental injury (TDI).

ASD severity	Study participant per group	
	TDI	No TDI
Requires support	2 (5%)	68 (17%)
Requires substantial support	24 (60%)	202 (50.4%)
Requires very substantial support	13 (32.5%)	128 (31.9%)
Missing data	1 (2.5%)	3 (0.7%)
Total	40	401

**TABLE 3:** Distribution of severity/type of traumatic dental injuries (TDI) by severity of autism spectrum disorder (ASD).

TDI severity/type	ASD severity		
	Requires minimal support	Requires substantial support	Requires very substantial support
Fracture limited to enamel		1 (4.2%)	1 (7.7%)
Fracture extending to dentin	1 (50%)	4 (16.7%)	5 (38.5%)
Fracture with pulp exposure		1 (4.2%)	
Fracture Crown to root		1 (4.2%)	
Concussion		8 (33.3%)	5 (38.5%)
Subluxation	1 (50%)	2 (8.3%)	
Subluxation with dentinal fracture			1 (7.7%)
Extrusive or lateral luxation		4 (16.7%)	
Extrusive or lateral luxation with dentinal fracture		1 (4.2%)	
Avulsion		2 (8.3%)	1 (7.7%)

*Note:* One participant with a fracture extending to dentin had an unknown ASD severity.

## 4 Discussion

While studies have shown that children with ASD may be at an increased risk of TDI [7], little is known about how the various features of ASD, including its severity, influence the risk. Understanding whether children with greater support needs are at greater risk of TDI is critical for guiding management strategies, resource allocation, and education on TDI prevention. The current study attempted to address this knowledge gap by examining the relationship between ASD severity and the presence and type/severity of TDI. To the authors' knowledge, this is the first study to examine the association between ASD severity and TDI severity and incidence.

Prior research indicates that children with more severe ASD are at an increased risk for poor dental health [7, 11, 17], which may increase the severity of TDI by weakening enamel and dentin, impairing periodontal ligament function, and disrupting occlusal stability, thereby reducing the teeth's resistance to trauma [22, 23, 24]. A higher level of decayed, missing and restored teeth has also been associated with a greater risk of TDI [15]. Furthermore, severe cases of ASD often involve cognitive and developmental delays that may reduce awareness of environmental hazards and impair sensory processing, thereby increasing the risk of TDI [25, 26, 27]. Although greater ASD severity has been assumed to increase the risk of TDI, this association was not observed in this study. A possible explanation is that children with more severe ASD may receive closer supervision from caregivers, or have reduced exposure to activities that increase the risk of TDI compared with those with milder cases of ASD [28].

Additionally, this study did not find a relationship between the severity of ASD and the type/severity of TDI. Studies have shown that individuals with more severe ASD, especially those with behavioral dysregulation, impulsivity, or self‐injurious tendencies, experience a higher incidence of severe TDI than neurotypical children [7, 18]. Children with ASD are often more likely to present with severe TDI, such as luxation, avulsion, or crown fracture extending into the pulp [14, 18, 29]. A systematic review reported that enamel and dentin fractures, subluxation, and lateral luxation occur more frequently in the ASD population [30]. Increased TDI severity in ASD may be attributed to behavioral factors, cognitive and developmental delays, motor deficits, or a higher prevalence of severe dental overjet [18].

Considering that the study population may influence the observed correlations, and that 76% of the study population was represented by boys, the impact of sex was evaluated. However, no significant association could be observed between sex and the incidence of TDI. This is contrary to the existing literature reporting a higher occurrence of TDI in females with ASD [14, 31]. This differs from the general population, where males are reported to be twice as likely to sustain TDI [32, 33]. However, girls with ASD are about 4 times more likely to remain undiagnosed and are often only diagnosed when their symptoms become more severe [34, 35]. Therefore, females diagnosed with ASD often need more supports, which was suggested as a potential rationale for the increased incidence of TDI observed in other studies. However, this connection was not seen in this study.

While ASD severity is categorized into three distinct levels [9], the broad heterogeneity of symptoms often prevents clear‐cut clinical presentations. Individuals may exhibit mild symptoms in certain categories and more severe impairments in others, highlighting the limitations of this categorical approach. It may be argued that the current ASD severity classification emphasizes impairments in communication and restricted‐repetitive behaviors rather than physical or behavioral aspects contributing to the TDI. However, although physical aspects are not explicitly included in the severity criteria, ASD severity levels may still serve as a useful proxy for the level of support required in daily activities and their association with the risk of dental trauma.

Study limitations must be considered when interpreting findings. First, the retrospective study design limited the completeness and consistency of the data as it relied on pre‐existing medical documents. Assessing the patient charts can sometimes be challenging because clinicians may not always systematically record all relevant details in a standardized way. It is also important to consider that only a small subset of the study population developed a TDI, reducing statistical power; longer follow‐ups and a larger and more heterogeneous sample may provide additional insights. Similarly, the proportion of patients in each ASD severity group was dissimilar and may have influenced the results. This may be explained because more severe cases may be more likely to be referred to receive care in specialized health centers, while milder cases may be undiagnosed or treated locally. Still, 16% of the sample consisted of milder cases, providing sufficient power for analyses. Lastly, while the health center serves patients from multiple regions, potentially mitigating sampling bias, confounding factors such as sex, comorbidities, and family socioeconomic status could influence the results which may not generalize to all populations.

Since the study results are limited by the relatively small sample size, the results should be considered with precaution and future research with larger sample sizes and longer follow‐ups should be used to confirm the findings. In future studies, a neurotypical control group may help clarify the relative risk of TDI in children with ASD. A prospective study design with standardized tools for assessing ASD severity and other dental, cognitive, socio‐demographics, emotional, and behavioural characteristics may help data interpretation. Investigating the causes of TDI, environmental risk factors, management, and treatment outcomes may also provide more insight into the causes of TDI and the effectiveness of their management, which may help health providers identify higher‐risk children with ASD, target preventive measures, educate families, and allocate resources to reduce TDI occurrence and severity.

## 5 Conclusion

Within the limitations of this retrospective study, ASD severity and sex were not statistically associated with the occurrence or severity of TDI among children with ASD. Additional unidentified risk factors may be associated with TDI incidence and severity. These should be assessed in further studies.

## Author Contributions

Parisa Ghanouni: Conceptualization, Methodology, Writing – Original Draft, Supervision, Project administration. Pierre‐Luc Michaud: Methodology, Formal analysis, Writing – Review and Editing, Supervision. Annika Nobbe: Investigation, Writing – Original Draft. Isabel Mello: Methodology, Writing – Review and Editing, Supervision. Shannon Fitzpatrick: Methodology. Erin Quigley: Methodology, Resources, Supervision. Jarrett Miller: Investigation. Tracy Doyle: Methodology, Resources, Writing – Original Draft, Supervision.

## Funding

The research was funded by the Faculty of Dentistry, Dalhousie University.

## Ethics Statement

The project received approval from the institutional Research Ethics Board (REB file #1029896) prior to debuting the study.

## Consent

The authors have nothing to report.

## Conflicts of Interest

The authors declare no conflicts of interest.

## Data Availability

The authors have nothing to report.
